# 

**Published:** 1831-10

**Authors:** 


### MONTHLY LIST OF MEDICAL BOOKS.

[*Medical Works cannot be entered on this List unless a copy be sent for the purpose, the titles of Books having frequently been sent to us as published which have not been published for weeks, or even months, after.*]

AUR. COR. CELSUS on Medicine, in eight Books, Latin and English. Translated from L. TARGA's Edition; the Words of the Text being arranged in the Order of Construction. To which are prefixed, a Life of the Author, Tables of Weights and Measures, with explanatory Notes, &c., designed to facilitate the Progress of Medical Students. By ALEXANDER LEE, A.M., Surgeon. In two volumes. Vol. I.—8vo. pp. 318. Cox, London.

Cholera; its Nature, Cause, Treatment, and Prevention, clearly and concisely explained. By CHARLES SEARLE, Esq., of the Hon. East India Company's Madras Establishment.—Pp. 38. Wilson, London.

Pathological Anatomy of the Brain, Spinal Cord, and their Membranes: being a condensed Description of the Morbid Appearances generally met with after Death; with Cases. Illustrated by thirteen coloured Plates. By W. P. COCKS, Surgeon.—18mo. pp. 175. Highley, London.

Principles of Lithotrity; or, a Treatise on the Art of Extracting the Stone without Incision. By Baron HEURTELOUP, Doctor of the Faculty of Medicine, Paris. Illustrated with Plates of the Instruments used in Lithotrity.—8vo. pp. 483. Whittaker, Treacher, and Co. London.

Lecture introductory to a Course of Clinical Surgery, delivered to the Students of the Glasgow Royal Infirmary, by M. S. BUCHANAN, M.D., Member of the Faculty of Physicians and Surgeons, Glasgow; and one of the Surgeons to the Royal Infirmary, &c.

An Account of a Contagious Fever which occurred amongst the Danish and American Prisoners of War at Chatham, in the Years 1813, 1814. By Sir WM. BURNETT, Knt., M.D., K.C.H., &c.—8vo. pp. 47. Burgess and Hill, London.

Observations and Experiments on a New Mode of Treating Fractures of the Leg and Fore Arm; especially Compound Fractures. By WILLIAM BEAUMONT, Member of the Royal College of Surgeons, and Surgeon to the Farringdon Dispensary.—8vo. pp. 31. Longman and Co., London.

### NOTICES.

Mr. MITCHELL's *Cases of Mercurial Palsy will be inserted in the next number.*

"A Surgeon" *is mistaken:* we have *very maturely deliberated on his proposal, and regret that we cannot avail ourselves of his assistance.*

*We shall be obliged to the learned Translator of* PAULUS ÆGINETA, *if he will favor us with a Prospectus of his Work: we have had several inquiries concerning it, and wish to lay before our Readers the plan of the Work.*

*The "rare cases" of our Devonshire Correspondent have really no rarity in them: he will find similar examples of the disease in every practical writer.*

Mr. ADAMS' *Paper, which we have received through* Mr. GUTHRIE, *on the "Opinions of the Ancients on the Causes and Treatment of Cholera," in our next number.*

*We are much obliged to our Correspondent from Bury St. Edmond's: his suggestion shall be attended to.*

